# Association Between Stroke and Traumatic Brain Injury: A Systematic Review and Meta-Analysis

**DOI:** 10.3390/neurosci6010021

**Published:** 2025-03-04

**Authors:** Mohammed Maan Al-Salihi, Maryam Sabah Al-Jebur, Ahmed Abd Elazim, Ram Saha, Ahmed Saleh, Farhan Siddiq, Ali Ayyad, Adnan I. Qureshi

**Affiliations:** 1 Zeenat Qureshi Stroke Institute, University of Missouri, Columbia, MO 65211, USA; 2 College of Medicine, University of Baghdad, Baghdad 00964, Iraq; 3 Department of Neurology, University of South Dakota, Sioux Falls, SD 57105, USA; 4 Department of Neurology, Virginia Commonwealth University, Richmond, VA 23298, USA; 5 Department of Neurosurgery, Hamad General Hospital, Doha 00974, Qatar; 6 Department of Neurosurgery, University of Missouri, Columbia, MO 65211, USA; 7 Department of Neurosurgery, Saarland University Hospital, 66421 Homburg, Germany; 8 Department of Neurology, University of Missouri, Columbia, MO 65211, USA

**Keywords:** traumatic brain injury, stroke, risk association, meta-analysis, systematic review

## Abstract

Background: Stroke and traumatic brain injury (TBI) represent two major health concerns worldwide. There is growing evidence suggesting a potential association between TBI and stroke. In this systematic review and meta-analysis, we aim to explore the association between TBI and stroke risk, with a specific focus on overall stroke risk and subgroup variations based on stroke type, severity, and the post-TBI time period. Methods: PubMed, Web of Science (WOS), Scopus, and Cochrane Library were systematically searched for studies exploring the link between stroke and TBI. The pooled hazard ratios (HRs) with a 95% confidence interval (CI) were calculated. The Comprehensive Meta-Analysis (CMA) software was used for the analysis. Subgroup analyses were conducted based on stroke type, TBI severity, and post-TBI phase. The Newcastle–Ottawa Scale (NOS) was utilized for the quality assessment. Results: We included a total of 13 observational studies, with data from 8 studies used for quantitative analysis. A history of TBI was associated with a significantly higher odds of stroke compared to controls (HR = 2.3, 95% CI (1.79 to 2.958), *p* < 0.001). The risk was greater for hemorrhagic stroke (HR = 4.8, 95% CI (3.336 to 6.942), *p* < 0.001) than for ischemic stroke (HR = 1.56, 95% CI (1.28 to 1.9), *p* < 0.001). Both moderate-to-severe TBI (HR = 3.64, 95% CI (2.158 to 6.142), *p* < 0.001) and mild TBI (HR = 1.81, 95% CI (1.17 to 2.8), *p* = 0.007) were associated with a significantly higher risk of stroke. The risk was also higher in the early post-TBI phase (1–30 days) (HR = 4.155, 95% CI (2.25 to 7.67), *p* < 0.001) compared to later phases (HR = 1.68, 95% CI (1.089 to 2.59), *p* = 0.019) from 30 days to 1 year and (HR = 1.87, 95% CI (1.375 to 2.544), *p* < 0.001) after 1 year. Conclusions: This systematic review confirms a significant association between TBI and an increased risk of stroke, regardless of TBI severity, type, or timing of stroke. The findings highlight the need for early monitoring and advocating preventive strategies for stroke in patients with a history of TBI.

## 1. Introduction

Each year, over 50 million people around the world suffer from TBI. This entails a wide range of injuries, from mild head trauma to more severe penetrating injuries [1]. It is known as one of the leading causes of mortality and long-term neurological complications, including cognitive impairment, post-traumatic epilepsy, increased risk for neurodegenerative diseases, and stroke [2,3]. Approximately 80% of cases are categorized as mild TBI. While the prognosis for mild TBI is generally favorable, outcomes worsen with older individuals, where the risk of mortality increases [4]. A growing body of evidence indicates a potential link between having a history of TBI and stroke. The combined incidence of stroke and TBI is substantial, given their serious complications, which may result in long-term disability and impose a significant economic burden on public health systems [5,6,7,8,9].

The link between stroke and TBI may be due to the different mechanisms related to post-TBI cerebral vasculature responses in terms of vasculopathy, changes in cerebral blood flow, and chronic inflammation [10]. However, vulnerability to stroke after TBI may vary based mainly on the severity and the timing after TBI. It is essential to evaluate the risk of stroke across different levels of TBI severity. Mild TBI represents 80% of all TBIs. Hence, special attention should be paid to quantitatively measure its associated stroke risk. Understanding these aspects is crucial for developing effective preventive measures, tailored to TBI survivors at a higher stroke risk.

Findings from a recent meta-analysis indicated a significant link between TBI and stroke, with a lower risk for ischemic stroke when compared to the overall association (ischemic or hemorrhagic) [11]. In the early post-TBI phase, two studies reported vastly different hazard ratios for stroke incidence within 1 month after TBI (1.7 versus 10.2) [4,12]. Over extended follow-up periods, the reported risks still varied significantly in the literature, ranging from 1.1 to 4.6 times. Furthermore, studies highlighted varying degrees of association between having a history of TBI and the occurrence of ischemic and hemorrhagic strokes. This meta-analysis aims to update the previous findings by incorporating more recent studies with large datasets to further enhance the robustness and precision of the risk estimates [12,13]. In this systematic review and meta-analysis, we aim to evaluate the association between TBI and stroke risk, with a specific focus on overall stroke risk and subgroup variations based on stroke type, severity, and the post-TBI time period.

## 2. Methods

This systematic review was compliant with the guidelines set by the Preferred Reporting Items for Systematic Reviews and Meta-Analyses (PRISMA) [14]. The PRISMA checklist for this meta-analysis is provided in the Supplementary Material.

### 2.1. Search Strategy and Selection Process

We searched through 4 databases, PubMed, Scopus, Web of Science, and the Cochrane Library, from their inception to 10 October 2024. Without applying any filters, the following terms were used (“Traumatic brain injury” OR “TBI” OR “Head trauma” OR “Head injuries” OR “Concussion”) AND (“Stroke” OR “Cerebrovascular accident” OR “Brain vascular accident” OR “Brain ischemia” OR “Non-ischemic stroke” OR “Ischemic stroke”) AND (“Risk factors” OR “Indicators” OR “Incidence” OR “Association” OR “Predictors”) (Table S1). Retrieved records were exported to Endnote to detect and remove the duplicates. All relevant studies were obtained and assessed for the predetermined inclusion and exclusion criteria. Two independent reviewers conducted this process, and any disagreements were resolved by consulting a third reviewer for consensus.

### 2.2. Eligibility Criteria

We included any study investigating the association between stroke and TBI. Regarding the population, we included studies with adult cases with a previous history of TBI with no restrictions in terms of mode of trauma or TBI severity (mild, moderate, or severe). Only double-arm studies with a control group were included. The primary outcome was the odds of stroke events (hemorrhagic or ischemic) at any time point after TBI.

### 2.3. Data Extraction and Risk of Bias

Two independent reviewers extracted the data from the included studies. Information on study characteristics, including sample size, study design, country, institution, recruitment period, follow-up period, and study conclusion, were extracted and tabulated in a summary table. Baseline data, including number of exposed (TBI) to non-exposed (non-TBI), mean age, gender distribution, and comorbidities, were extracted and tabulated in another table. Available effect estimates (hazard ratios or rate ratios) of overall stroke, hemorrhagic stroke, and ischemic stroke at various time points after TBI and different levels of TBI severity were sought to be exported into an Excel sheet for analysis. We used the Newcastle–Ottawa Scale (NOS) as a tool used for quality assessment, evaluating studies across three main categories: selection, comparability, and outcome assessment. Each study was assigned a rating of “Good”, “Fair”, or “Poor” [15].

### 2.4. Statistical Analysis

The analysis was conducted using Comprehensive Meta-Analysis (CMA) version 3 software. The pooled hazard ratios (HRs) were obtained. Two studies reported the effect estimate as rate ratio and were not included in the analysis [5,9]. A random-effects model was employed in all analyses to consider the variability among the included studies. Subgroup analyses were conducted based on type of stroke, post-TBI phases, and severity of TBI. Heterogeneity was evaluated using the I^2^ statistic, with values over 50% and a *p* value below 0.1 indicating significant heterogeneity [16]. Egger’s test and funnel plots were applied to evaluate publication bias; asymmetrical funnel plots or a significant result from Egger’s test would suggest the presence of potential publication bias [17].

## 3. Results

### 3.1. Search Results

**Figure 1:**
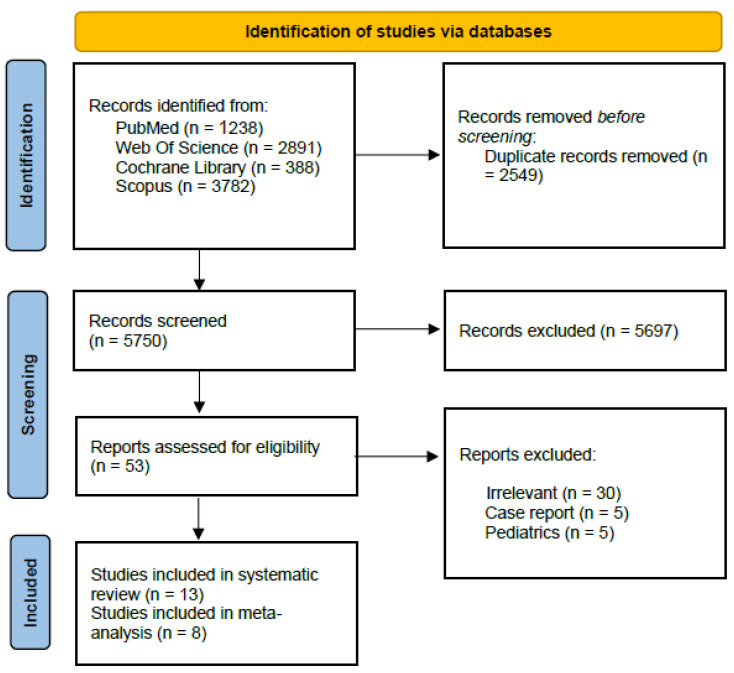
PRISMA flowchart.

Our search identified 8299 records, with 2549 being duplicates. A total of 53 studies were retrieved. Out of them, 13 studies met our pre-specified criteria and were included in this systematic review [4,5,6,8,9,12,13,18,19,20,21,22,23], with 5 studies not included in the analysis [5,6,8,9,22]. The PRISMA flowchart is shown in Figure 1.

### 3.2. Study Characteristics and Narrative Synthesis

All studies included in this systematic review were observational cohort studies. Patients were recruited from different databases worldwide across different timelines, ranging from 1987 to 2019: five studies were conducted in Taiwan, using their health insurance database [4,6,8,21,22], seven studies were located in the USA [5,9,13,18,19,20,23], and one study was conducted in Korea [12]. Due to the potential overlap in data across the five studies from Taiwan, we included only two studies with distinct, non-overlapping durations in the meta-analysis [4,21]. Follow-up durations ranged from 2 to 19 years, with a mean duration of 6.7 years. The mean ages in two studies were the lowest among all included studies: Choi et al. and Stewart et al. [12,23]. The former focused on the risk of stroke in young-to-middle-aged adults with a history of TBI, and the second focused on the association between TBI and the risk of cardiovascular diseases in post-9/11 military veterans. Some studies focused on the overall association between TBI and stroke. Others investigated different factors, like TBI severity and type of stroke. Some studies explored the association between concussion, the mild form of TBI, and stroke outcomes. Two studies focused on specific populations (U.S. veterans) and explored the long-term cardiovascular risks associated with TBI [13,23]. The characteristics and baseline data of the included studies are summarized in Table 1 and Table 2.

Two out of the five excluded studies from the meta-analysis reported their effect estimates as rate ratios rather than hazard ratios. Additionally, the study by Albrecht and colleagues did not include a healthy separate control group; otherwise, all cases had TBI, and they compared incidence of stroke among patients who experienced it before and after TBI [5]. Hence, we decided not to pool the rate ratio reported in this study with the one reported in McFarlane and colleagues’ study [9]. Albrecht and colleagues retrospectively analyzed 16,936 cases with a previous history of TBI, reporting that TBI significantly increased the risk of hemorrhagic stroke by 6-fold (rate ratio (RR) = 6.5, 95% CI, 5.3 to 7.8). Additionally, a smaller increase in the odds of ischemic stroke was reported (RR = 1.3, 95% CI, 1.2 to 1.4). McFarlane and colleagues analyzed the incidence of acute ischemic stroke in 58,294 TBI cases. They reported that the risk was higher in early post-TBI phases: 2.3% in the first month, 0.31% after the first month, and 0.13% after six months. They concluded that acute and post-acute stages of TBI had a higher risk of acute ischemic stroke, especially among younger ages, cervical artery dissection, and severe TBI.

The other three studies, Nyam et al., Liao et al. and Liu et al. [6,8,22], were excluded from the meta-analysis due to potential data overlap with each other and with the studies by Chen et al. and Lee et al. that were already included [4,21]. These studies used data from Taiwan’s National Health Insurance Research Database with overlapping study periods, which could lead to duplicated data points across analyses. By excluding these studies, we aimed to minimize redundancy and potential biases that could otherwise skew the meta-analysis results. This approach ensures that the findings represent unique patient groups and maintains the validity of our pooled estimates. Liao and colleagues analyzed a total of 30,165 TBI cases and 120,660 individuals without TBI from Taiwan’s National Health Insurance Research Database from 2000 to 2004, monitoring stroke risk through to 2008. They found that TBI patients had a significantly higher risk of stroke (HR = 1.98) than those without TBI. Furthermore, TBI severity was correlated with post-stroke mortality, highlighting the need for targeted prevention [22]. Liu and colleagues analyzed data of 13,652 concussion cases from the same registry from 1998 to 2005. This concussion group was then compared to an equivalent group matched by sex, age, and propensity score. Stroke risk was notably elevated in those with concussion, with both crude and adjusted hazard ratios indicating a heightened risk. Similarly, Nyam and colleagues reviewed data from the same registry from 2000 to 2012. They reported that TBI had increased the risk of major adverse cardiac and cerebrovascular events (MACCEs) by 2.77-fold. The risk of hemorrhagic stroke alone increased by 6-fold [6].

### 3.3. Meta-Analysis

#### 3.3.1. Stroke Risk After TBI Based on Stroke Type

**Figure 2:**
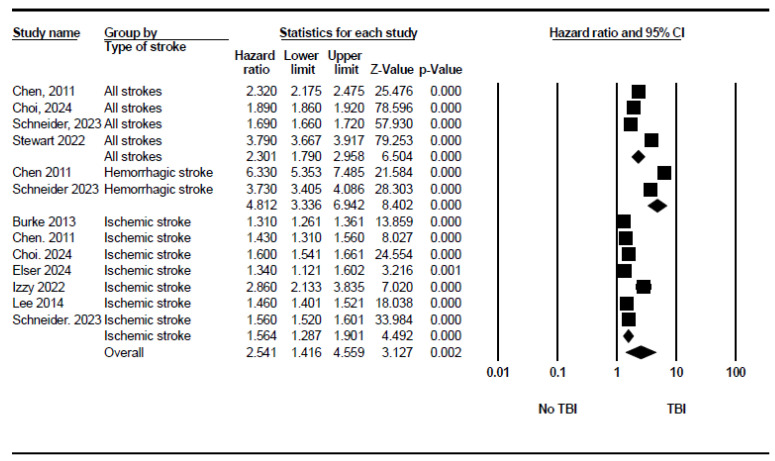
Forest plot showing the pooled stroke risk after TBI based on the stroke type.

The pooled analysis of four studies demonstrated that a history TBI significantly increased the overall risk of stroke compared to those without a TBI history (HR = 2.3, 95% CI: 1.79 to 2.96, *p* < 0.001). Two out of these four studies reported the isolated hazard ratios of hemorrhagic stroke. The pooled analysis of them showed around a 5-fold increase in the risk of hemorrhagic stroke in the TBI group (HR = 4.81, 95% CI: 3.34 to 6.94, *p* < 0.001). Seven studies reported the isolated hazard ratios of ischemic stroke. Similarly, TBI was associated with significantly higher odds of ischemic stroke but with a lower hazard ratio than of hemorrhagic stroke (HR = 1.56, 95% CI: 1.29 to 1.90, *p* < 0.001). Figure 2 demonstrates the forest plot of these subgroups. The between-subgroup heterogeneity in the random-effects model was significant, with I^2^ = 93.07% and *p* < 0.001. This suggests that the magnitude of the increased risk of stroke associated with TBI varies significantly depending on the stroke subtype. Egger’s test for publication bias showed an intercept of 4.89 (95% CI: −12.03 to 21.82), indicating no significant publication bias (Supplementary Figure S1).

#### 3.3.2. Stroke Risk After TBI Based on TBI Severity

**Figure 3:**
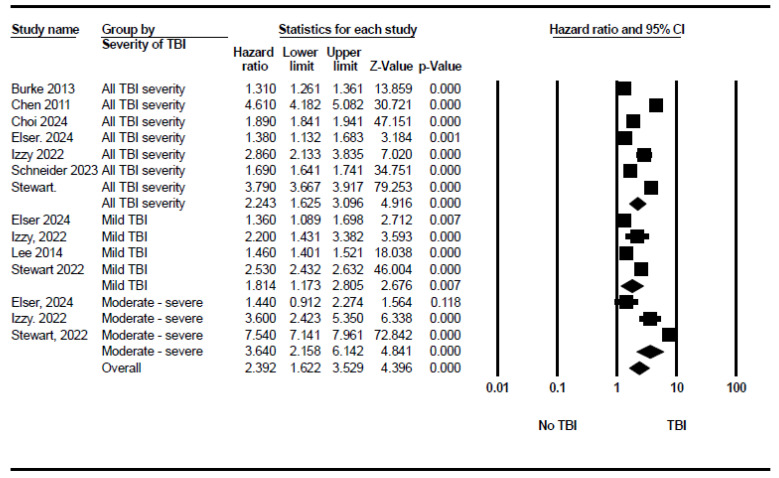
Forest plot showing the pooled stroke risk after TBI based on stroke severity.

The pooled analysis of seven studies demonstrated that TBI, whatever the severity, significantly increased the overall risk of stroke (HR = 2.243, 95% CI: 1.625 to 3.096, *p* < 0.001). A total of four studies reported the isolated hazard ratios of stroke after mild TBI (concussion). The pooled analysis showed an increase in the risk of stroke after mild TBI (HR = 1.814, 95% CI: 1.173 to 2.805, *p* = 0.007). A total of three studies reported the hazard ratios of stroke after moderate-to-severe TBI, showing a higher risk (HR = 3.640, 95% CI: 2.158 to 6.142, *p* < 0.001). Figure 3 demonstrates the forest plot of these subgroups. The test for heterogeneity between subgroups in the random-effects model showed an I^2^ of 51.4% and a *p* value of 0.128, indicating non-significant heterogeneity. Egger’s test for publication bias yielded an intercept of 3.90 (95% CI: −14.90 to 22.69), suggesting no significant publication bias (Supplementary Figure S2).

#### 3.3.3. Stroke Risk After TBI Based on Post-TBI Phase

**Figure 4:**
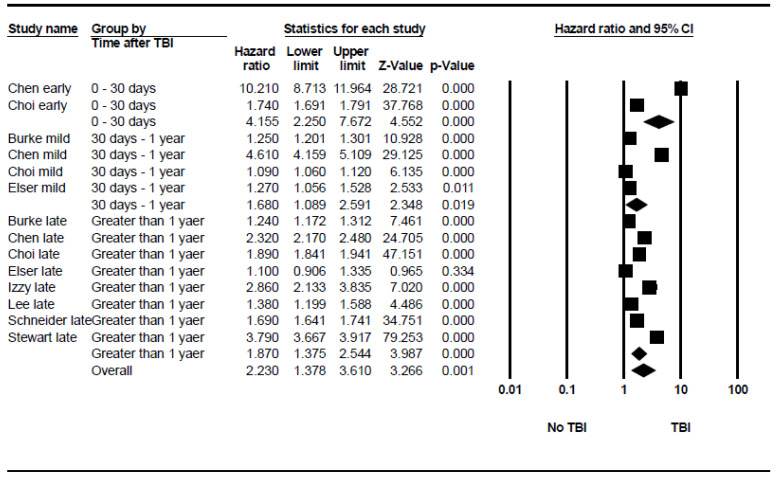
Forest plot showing the pooled stroke risk after TBI based on post-TBI phase.

Pooling the estimates from two studies, the risk of stroke was highest within the first month post-TBI (HR = 4.155, 95% CI: 2.25 to 7.67, *p* < 0.001). The hazard ratio decreased with time to (HR = 1.68, 95% CI: 1.089 to 2.591, *p* = 0.019) and (HR = 1.87, 95% CI: 1.375 to 2.544, *p* < 0.001) within the durations of 30 days to 1 year and after 1 year, respectively. Between-group differences were significant. Figure 4 demonstrates the forest plot of these subgroups. The between-subgroup heterogeneity in the random-effects model was moderate to high, with I^2^ = 67.98% and *p* value = 0.044. This suggests that the risk effect sizes vary across the subgroups defined by different time periods. Egger’s test for publication bias showed an intercept of 5.79 (95% CI: −14.43 to 26), indicating no significant evidence of publication bias (Supplementary Figure S3).

### 3.4. Risk of Bias and Certainty of Evidence

Using the NOS, most included studies were of good quality, scoring ≥9 due to robust methodologies, including adequate cohort selection, comparability, and outcome assessment. However, two studies [5,9] were rated as fair quality, with lower scores in the comparability and outcome domains due to limited follow up, inadequate exposure ascertainment, and cohort comparability. The detailed results of the quality assessment are presented in Table S2. Using the GRADE (Grading of Recommendations, Assessment, Development, and Evaluations) assessment, we found low-certainty evidence for the association between TBI and stroke across different subgroups, including stroke type, TBI severity, and post-TBI phases. This is because all the included studies were observational, which inherently limits the certainty of evidence. Detailed results of the quality assessment are presented in Table S3.

## 4. Discussion

This meta-analysis supported the evidence of the significant association of TBI with an increased risk of stroke regardless of stroke type, TBI severity, and post-TBI phase. Notably, the hazard ratios were higher for hemorrhagic stroke than ischemic stroke (HR = 4.8 vs. 1.56), for moderate-to-severe TBI than mild TBI (HR = 3.64 vs. 1.8), and for early post-TBI phase than late phases (HR = 4.155 vs. 1.68 vs. 1.87). Findings from subgroup analyses may provide more individualized risk stratification, allowing for the implementation of preventive strategies or close follow up of such patients, with more meticulous attention for patients with severe TBI and within 1 month after any TBI.

Two previous meta-analysis studies have pooled the evidence regarding this association. Turner and colleagues included 18 studies. However, only six studies had non-TBI control groups. They reported a significant association between TBI and stroke with a pooled hazard ratio of 1.86 (95% CI 1.46 to 2.37). Additionally, their findings suggested that stroke risk is highest in the first four months after TBI but remains significantly high for up to five years [24]. A more recent meta-analysis by Esterov and colleagues reported similar findings with a slightly higher hazard ratio (HR = 2.06, 95% CI, 1.28 to 3.32). They also reported significant differences in study subgroups, noting that the risk for ischemic stroke was lower than for all stroke types combined [11]. Our meta-analysis builds upon these findings by including more recent studies in the overall and subgroup analyses. The previous evidence about the risk of stroke after 1 year of TBI was not conclusive. However, our meta-analysis included data from nine studies, reporting follow up after 1 year. The hazard ratio continued to be significantly high (HR = 1.963, 95% CI: 1.478 to 2.608). Notably, the risk wanes with time, as reported by Chen et al. and Choi et al. [4,12].

The stroke attributed to TBI is described as a secondary brain injury, and this association can be explained by multiple interrelated mechanisms, predominantly involving vascular changes, blood–brain barrier (BBB) dysregulation, cerebrovascular dysregulation, and neuroinflammatory responses [10,25]. The BBB disruption leads to increased permeability, allowing neurotoxic substances, immune cells, and inflammatory mediators to infiltrate the brain parenchyma. This breach triggers neuroinflammation, a process that begins early after TBI and involves a cascade of metabolic and immunologic reactions, including microglial activation and the release of pro-inflammatory cytokines such as interleukin-1β and tumor necrosis factor-α, ultimately leading to endothelium damage, which exacerbates vascular instability. This increases the likelihood of hemorrhagic stroke due to the weakening of cerebral blood vessels. Additionally, this may increase the risk of ischemic stroke since these pro-inflammatory cytokines can promote endothelial activation and dysfunction, increasing the risk of ischemic stroke by facilitating thrombus formation and vascular occlusion. Furthermore, these inflammatory responses impair cerebral autoregulation and alter vascular reactivity, which can result in vasospasm or hypoperfusion. These conditions predispose the brain to ischemic injury, especially in the setting of additional systemic insults like hypotension or hypoxia. The autoregulatory mechanism proved to be highly sensitive to trauma or injury, whether resulting from mild or severe TBI. An increase in intracranial pressure and brain swelling, which lead to a reduction in cerebral perfusion pressure (CPP), are believed to disrupt autoregulation [26,27,28].

Post-TBI hypercoagulability can be attributed to platelet hyperactivity, increased expression of tissue factor, and early disseminated intravascular coagulation [29]. The process of neuroinflammation begins early after TBI, involving a cascade of metabolic and immunologic reactions and ultimately leading to secondary neural death [30]. Those changes can be affected by some factors. Studies have identified different baseline factors that made patients with moderate-to-severe TBI more prone to stroke. These factors included a low baseline Glasgow Coma Scale score, low blood pressure, respiratory issues on admission, carotid or vertebral artery dissection, brain herniation, and operative interventions [28,31,32,33]. Further studies suggested that antipsychotic and antidepressant drugs taken for TBI-related psychiatric disorders can play a role in increased stroke risk [5,34].

All mentioned factors and mechanisms can explain the well-established association between stroke and TBI during the acute and subacute periods. However, the primary mechanisms underlying the long-term elevated risk of stroke following TBI remain unclear and require further investigation. As demonstrated in our meta-analysis, the increased risk of stroke continues over 1 year after the TBI event as persistent post-TBI inflammation leads to remodeling and stiffening of blood vessels, further impairing cerebral perfusion and increasing stroke susceptibility over time. Additionally, shared risk factors for TBI and stroke, such as undetected comorbidities and changes in health behaviors like reduced physical activity or the cessation of stroke-preventive medications, may partially account for this association [19]. Structural changes in the vasculature, such as microaneurysm formation, further elevate the risk of hemorrhagic events [35]. Choi and colleagues suggested that vascular wall damage or increased risk of amyloid angiopathy after TBI may increase vulnerability to delayed hemorrhagic or ischemic stroke [12]. Together, these processes—mediated by local and systemic inflammation and compounded by behavioral and physiological changes—underscore the complex interplay between TBI and delayed stroke development.

Another area that still needs more clarification is the mechanisms and factors associated with the higher stroke risk after mild TBI. Mild TBI may have mechanisms that are somewhat different from those operating in moderate and severe injuries. Neuroinflammation, a derangement in cerebral autoregulation, and microvascular damage are postulated to contribute to stroke risk even in mild cases [8,36]. The lower hazard ratio observed in the mild TBI subgroup compared to moderate or severe TBI is logical, as the pathophysiological changes following mild TBI, such as neuroinflammation, blood–brain barrier disruption, and cerebrovascular dysregulation, are expected to be less severe and less persistent. This reduced severity of post-TBI alterations could explain the lower risk of stroke in these patients. Additionally, we acknowledge that potential confounding factors, such as baseline comorbidities, pre-existing vascular risk factors, and differences in healthcare access or follow up, may also contribute to the observed differences in stroke risk between subgroups. Patients experiencing repeated mild TBIs (concussion), such as athletes or military personnel, may have compounding cerebrovascular and neuroinflammatory damage, heightening their vulnerability to ischemic events. Similarly, younger individuals with mild TBI could face prolonged exposure to these risks over their lifetime, particularly if underlying factors such as hypercoagulability or hypertension remain unaddressed. This highlights the need for targeted surveillance in mild TBI patients, focusing on vascular risk factors like hypertension and diabetes in older adults, and periodic imaging for younger or high-risk patients who are exposed to repeated concussions [19,20,21,23]. These findings underscore the long-term impact of mild TBIs, supporting public health efforts to prevent head injuries and promote tailored preventive strategies to reduce stroke risk.

The findings of this meta-analysis may guide future risk stratification tools that can be used to decide for whom we need to implement preventive measures. These measures should include the early identification of stroke risk, controlling risk factors, addressing post-TBI inflammation and hypercoagulability, and close follow up. Future studies should focus on this issue and study the efficacy of different pharmacologic interventions like statins, antithrombotic therapy, and others [24,34]. The elevated risk of hemorrhagic stroke, which is nearly fivefold compared to ischemic stroke, emphasizes the need for tailored preventive approaches. Specifically, while antithrombotic therapy might mitigate the risk of ischemic stroke, its use must be carefully considered given the increased risk of hemorrhagic stroke, particularly in the early post-TBI phase. The higher hazard ratios observed within the first month post-TBI further underscore the importance of close monitoring during this period. Identifying high-risk subgroups, such as those with moderate-to-severe TBI or vascular comorbidities, could guide the implementation of targeted interventions. Future studies are needed to refine risk stratification models and assess the safety and efficacy of antithrombotic therapy in these patients, balancing stroke prevention with the risk of intracranial bleeding. The general recommendations to mitigate the risk of stroke in patients with a history of TBI include active management of the modifiable risk factors of stroke, patient education, and close follow up for high-risk patients [12].

The strengths of this meta-analysis lie in its comprehensive search strategy and robust statistical approach, providing valuable insights into post-TBI stroke risk. However, several limitations must be acknowledged. Differences in healthcare access, stroke diagnostic criteria, severity criteria, and follow-up durations may significantly influence risk estimates. Limited access to care can delay diagnosis and treatment, while inconsistent diagnostic criteria may lead to variable case identification. Shorter follow-up periods may miss delayed strokes, whereas longer durations risk attributing unrelated events to TBI. These factors underscore the need for standardized methods and equitable access to ensure accurate estimates. Additionally, the retrospective design of all the included studies introduces potential biases. Heterogeneity across studies, stemming from variations in methodologies, definitions of TBI, and follow-up durations, was only partially addressed by the random-effects model. The lack of adjustment for baseline characteristics, such as age and comorbidities, further represents a key limitation in our analysis. Lastly, the analysis was limited to hazard ratios, leading to the exclusion of two U.S. studies that reported rate ratios. While this decision may have affected the overall power of the analysis, the differences in effect estimates between hazard ratios and rate ratios necessitated their exclusion to maintain consistency.

## 5. Conclusions

Results from this meta-analysis indicate that there is a significant non-negligible risk of both hemorrhagic and ischemic stroke after a traumatic brain injury of any severity and at any phase. The hazard ratios are higher for hemorrhagic stroke, moderate-to-severe TBI, and early post-TBI phase within 1 month. However, the risk was persistently significant for ischemic stroke, mild TBI, and in late post-TBI phases. This may help inform future recommendations on TBI-related stroke prevention, particularly for high-risk groups. Secondary prevention strategies should be considered in all TBI patients, giving more aggressive attention to those patients with moderate-to-severe TBI within the first month after TBI. Follow up should be continued for years for fear of late stroke. More studies are needed to confirm the factors associated with higher risks and to optimize the preventive measures and follow up.

## Figures and Tables

**Table 1:** Summary of the included studies.

ID	Study Design	Country	Institution	Recruitment Period	Follow-up Period	Total Population (n)	Primary Outcome	Conclusion
Albrecht 2015 [5]	Cohort	United States	5% sample of Medicare claims data	2006 to 2009	1 year	16,936	To estimate the incidence rates of hemorrhagic and ischemic stroke after hospital discharge for TBI in adults aged 65 years or older and compare them with pre-TBI rates.	Future studies should explore the causes of increased stroke risk after TBI and identify effective treatment options to mitigate stroke risk and enhance outcomes in older adults post-TBI.
Burke 2013 [18]	Cohort	United States	State Inpatient Databases (SIDs), State Emergency Department Databases (SEDDs), Healthcare Cost and Utilization Project (HCUP), and Agency for Healthcare Research and Quality	2005 to 2009	2 years	1,173,353	To explore whether TBI may be a risk factor for subsequent ischemic stroke.	TBI is associated with ischemic stroke, independent of other major predictors.
Chen 2011 [4]	Cohort	Taiwan	Longitudinal Health Insurance Database 2000 (LHID 2000)	2001 to 2003	5 years	92,796	To estimate the risk of stroke during a period of 5 years following a TBI, compared with individuals who did not suffer TBI during the same period.	This is the first study to report an increased risk of stroke in individuals who have experienced a TBI.
Choi 2024 [12]	Cohort	Korea	The Korean National Health Insurance Service (NHIS)	2008 to 2017	7 years	1,036,846	To evaluated stroke risk in young-to-middle-aged adults based on TBI severity and stroke subtypes.	Stroke prevention should remain a priority even for young-to-middle-aged adults with TBI.
Elser 2024 [19]	Cohort	United States	The ARIC study (Atherosclerosis Risk in Communities)	1987 to 2019	7.5 years	12,813	To examine the association of head injury with physician-adjudicated acute ischemic stroke in a community-based sample of U.S. adults.	In this community-based cohort, head injury was associated with subsequent ischemic stroke.
Izzy 2022 [20]	Cohort	United States	Mass General Brigham (MGB) Research Patient Data Registry (RPDR)	2000 to 2015	Up to 10 years	13,053	To evaluate the incidence of cardiovascular, endocrine, neurological, and psychiatric comorbidities in patients with mild or moderate-to-severe TBI and examine the associations between post-TBI comorbidities and mortality.	Findings indicate that TBI, regardless of severity, is linked to an increased risk of chronic cardiovascular, endocrine, and neurological comorbidities in patients without pre-existing diagnoses.
Lee 2014 [21]	Cohort	Taiwan	The National Health Insurance (NHI)	2007 to 2010	1.94 (1.18)	744,716	To determine whether patients in Taiwan with mild TBI have a higher risk of stroke compared to the general population.	Mild TBI is an independent significant risk factor for ischemic stroke.
Liao 2014 [22]	Cohort	Taiwan	Taiwan’s National Health Insurance Research Database	2000 to 2004	4 years	150,825	To examine whether patients with TBI have a higher risk of stroke or increased post-stroke mortality.	TBI was associated with risk of stroke and post-stroke mortality.
Liu 2017 [8]	Cohort	Taiwan	The National Health Insurance (NHI)	1998 to 2005	4 years	27,304	To clarify the association between concussion and stroke.	Concussion is an independent risk factor for both ischemic and hemorrhagic stroke.
McFarlane 2020 [9]	Cohort	United States	Indiana Network for Patient Care	2005 to 2014	10 years	58,294	To measure the risk of acute ischemic stroke after TBI based on its severity.	The acute and post-acute stages of TBI contribute to an increased risk of acute ischemic stroke, especially in younger patients, those with cervical artery dissection, and cases of severe TBI.
Nyam 2019 [6]	Cohort	Taiwan	Longitudinal Health Insurance Database (LHID)	2000 to 2012	5 years	48,633	To assess the long-term risk of all-cause major adverse cardiovascular events in a large, representative cohort of patients with TBI compared to matched controls, utilizing this nationwide database.	Patients with TBI have a significantly greater risk of major adverse cardiovascular and cerebrovascular events than the control group.
Schneider 2023 [13]	Cohort	United States	Veterans’ Health Administration (VHA) system	2002 to 2019	Median follow-up of 5.2 years	613,592	To analyze the long-term relationship between TBI and stroke and explore potential variations based on age, sex, race, ethnicity, and time since TBI diagnosis.	Veterans with prior TBI are at increased long-term risk for stroke, suggesting they may be an important population to target for primary stroke prevention measures.
Stewart 2022 [23]	Cohort	United States	U.S. Department of Veterans Affairs (VA) and Department of Defense (DoD) Identity Repository (VADIR), the VA Corporate Data Warehouse (CDW), the DoD and VA Infrastructure for Clinical Intelligence (DaVINCI), the Theater Data Management Store (TMDS), the DoD Trauma Registry (DoDTR), and the National Death Index (NDI)	1999 to 2016	19 years	1,559,928	To assess the relationship between TBI and the risk of developing cardiovascular diseases in post-9/11-era veterans.	The findings of this cohort study indicate that U.S. veterans with a history of TBI had a higher likelihood of developing cardiovascular diseases compared to those without a TBI history.

TBI; traumatic brain injury, mean (standard deviation).

**Table 2:** Baseline characteristics of the included studies.

ID	Number		Age (Years), Mean (SD)		Female Sex, n (%)		Diabetes, n (%)		Hypertension, n (%)		Chronic Kidney Disease, n (%)		Atrial Fibrillation, n (%)		Coronary Artery Disease, n (%)		Hyperlipidemia, n (%)	
	TBI	No TBI	TBI	No TBI	TBI	No TBI	TBI	No TBI	TBI	No TBI	TBI	No TBI	TBI	No TBI	TBI	No TBI	TBI	No TBI
Albrecht 2015 [5]	16,936	*	81.0 (7.9)	*	10,557 (6.2)	*	6535 (39)	*	NA	*	NA	*	4664 (28)	*	NA	*	NA	*
Burke 2013 [18]	436,630	736,723	49.2 (22.4)	50.3 (20.1)	204,298 (46.8)	363,210 (49.3)	31,897 (7.3)	59,141 (8.0)	75,438 (17.3)	125,752 (17.1)	7386 (1.7)	13,538 (1.8)	12,359 (2.8)	14,702 (2.0)	16,573 (3.8)	24,930 (3.4)	20,873 (4.8)	41,206 (5.6)
Chen 2011 [4]	23,199	69,597	41.6 (18.4)		10,768 (46.4)	32,304 (46.4)	2037 (8.8)	4836 (7.0)	3802 (16.4)	9916 (14.3)	NA	NA	99 (0.4)	199 (0.3)	1808 (7.8)	4344 (6.2)	1767 (7.6)	5342 (7.7)
Choi 2024 [12]	518,423	518,423	36 (11.85)	36 (11.85)	248,445 (47.92)	248,445 (47.92)	20,862 (4.02)	16,698 (3.22)	NA	NA	NA	NA	NA	NA	NA	NA	NA	NA
Elser 2024 [19]	2158	10,655	56.0 (6.67)	54.0 (7.41)	1419 (65.8)	5979 (56.1)	263 (12.2)	1286 (12.1)	798 (37.0)	3767 (35.4)	NA	NA	NA	NA	88 (4.1)	535 (5.0)	NA	NA
Izzy 2022 [20]	8702	4351	46.0 (20.74)	46 (20.74)	3910 (45)	1955 (45)	0	0	0	0	0	0	0	0	0	0	0	0
Lee 2014 [21]	24,905	719,811	46.1 (20.1)	43.5 (16.3)	13,091 (52.6)	370,830 (51.5)	2466 (9.9)	51,555 (7.2)	4936 (19.8)	111,872 (15.5)	NA	NA	133 (0.5)	2654 (0.4)	3878 (15.6)	78,417 (10.9)	2849 (11.4)	71,010 (9.9)
Liao 2014 [22]	30,165	120,660	44.5 (17.8)	43.9 (17.3)	14,936 (49.6)	59,852 (49.6)	3592 (11.9)	10,826 (9.0)	7398 (24.5)	26,592 (22.0)	353 (1.2)	799 (0.7)	NA	NA	NA	NA	3826 (12.7)	13,906 (11.5)
Liu 2017 [8]	13,652	13,652	56.3 (12.1)	56.2 (12.0)	7385 (54.1)	7373 (54.0)	1280 (9.4)	1249 (9.1)	2705 (19.8)	2689 (19.7)	NA	NA	NA	NA	1143 (8.4)	1145 (8.4)	NA	NA
McFarlane 2020 [9]	58,294	**	>18 years	**	29,670 (50.9)	**	11,342 (19.5)	**	24,157 (41.4)	**	NA	**	NA	**	11,360 (19.5)	**	NA	**
Nyam 2019 [6]	16,211	32,422	NA	NA	6382 (39.37)	12,746 (39.31)	1339 (8.26)	2577 (7.95)	2467 (15.22)	5173 (15.96)	250 (1.54)	558 (1.72)	NA	NA	NA	NA	NA	NA
Schneider 2023 [13]	306,796	306,796	50.2 (17.6)	50.4 (17.6)	27,866 (9.0)	27,866 (9.0)	46,088 (15.0)	36,557 (11.9)	11,595 (37.7)	94,700 (30.8)	NA	NA	13,256 (4.3)	6789 (2.2)	24,836 (8.1)	19,055 (6.2)	105,547 (34.4)	89,413 (29.1)
Stewart 2022 [23]	301,169	1,258,759	27 (8.15)	29 (11.11)	35,898 (11.9)	246,600 (19.6)	4388 (1.5)	25,481 (2.0)	32,220 (10.7)	14264 (11.3)	1047 (0.4)	5282 (0.4)	NA	NA	NA	NA	37,598 (12.5)	19,284 (15.3)

TBI; traumatic brain injury, SD; standard deviation, n; number, NA; not available. * No direct referent group; comparison of incidence rates: stroke before TBI vs. stroke after TBI. ** No direct reference group specified. The 2014 NHIS reported age-specific rates of ischemic stroke in the general population.

## Data Availability

All materials related to this meta-analysis, including data collection forms, extracted data, and analysis files, can be made publicly available upon request.

## Supplementary Materials

The following supporting information can be downloaded at: https://www.mdpi.com/article/10.3390/neurosci6010021/s1, Figure S1. Funnel plot assessing publication bias in the meta-analysis of stroke risk after traumatic brain injury (TBI).; Figure S2: Funnel plot assessing publication bias in the meta-analysis of stroke risk after traumatic brain injury (TBI) based on TBI severity.; Figure S3: Funnel plot assessing publication bias in the meta-analysis of stroke risk after traumatic brain injury (TBI) based on post-TBI phase.; Table S1: Summary of the used search strategy and results.; Table S2: Quality assessment of cohort studies using NOS tool.; Table S3: GRADE assessment.
