# Supplementary material for: Association Between Stroke and Traumatic Brain Injury: A Systematic Review and Meta-Analysis
Source: NeuroSci. 2025 Mar 4;6(1):21. doi: 10.3390/neurosci6010021 (PMC11944542; doi:10.3390/neurosci6010021)
Supplement: Supplementary file 1 [file neurosci-06-00021-s001.zip › neurosci-3427401-supplementary.pdf]

Figure S1

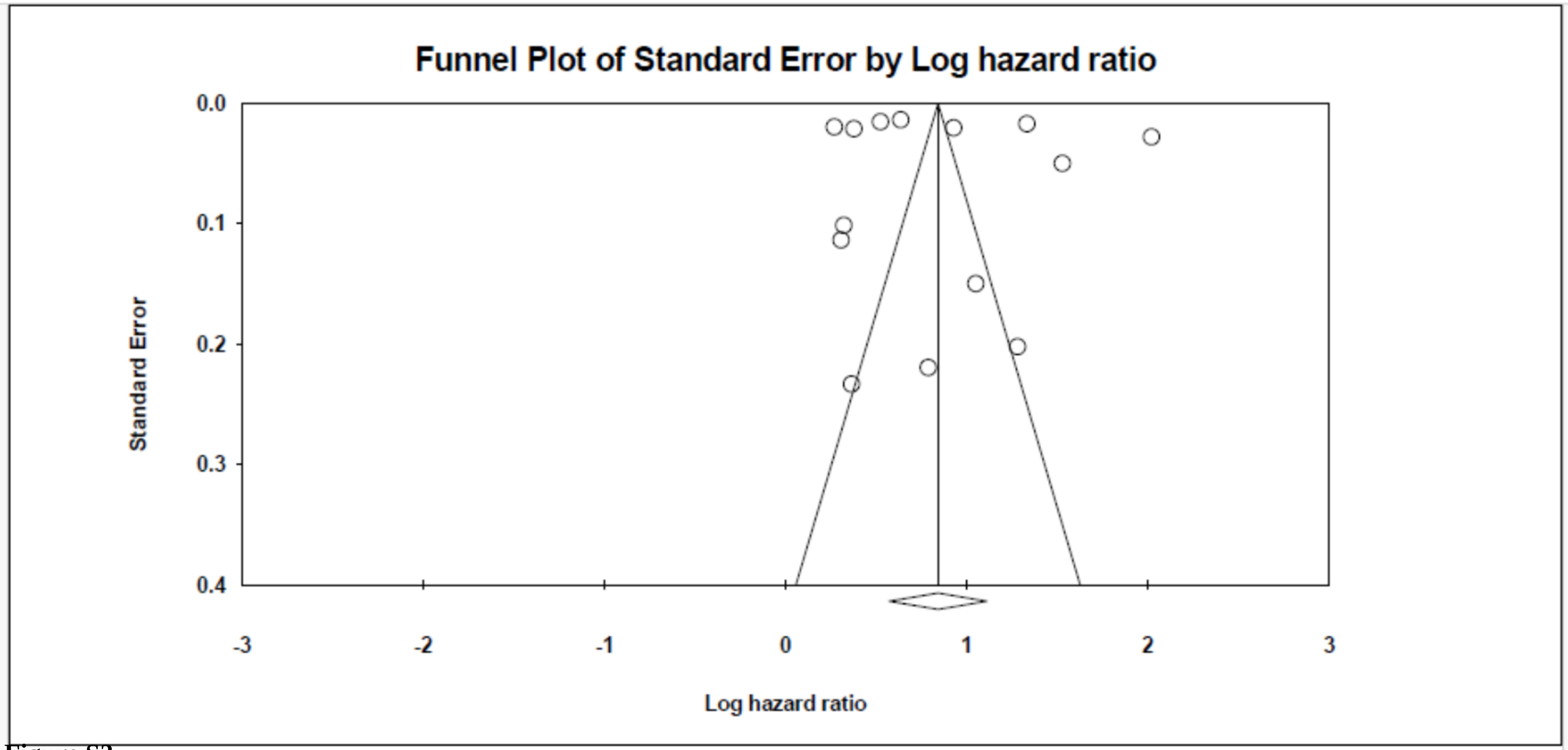

Figure S2

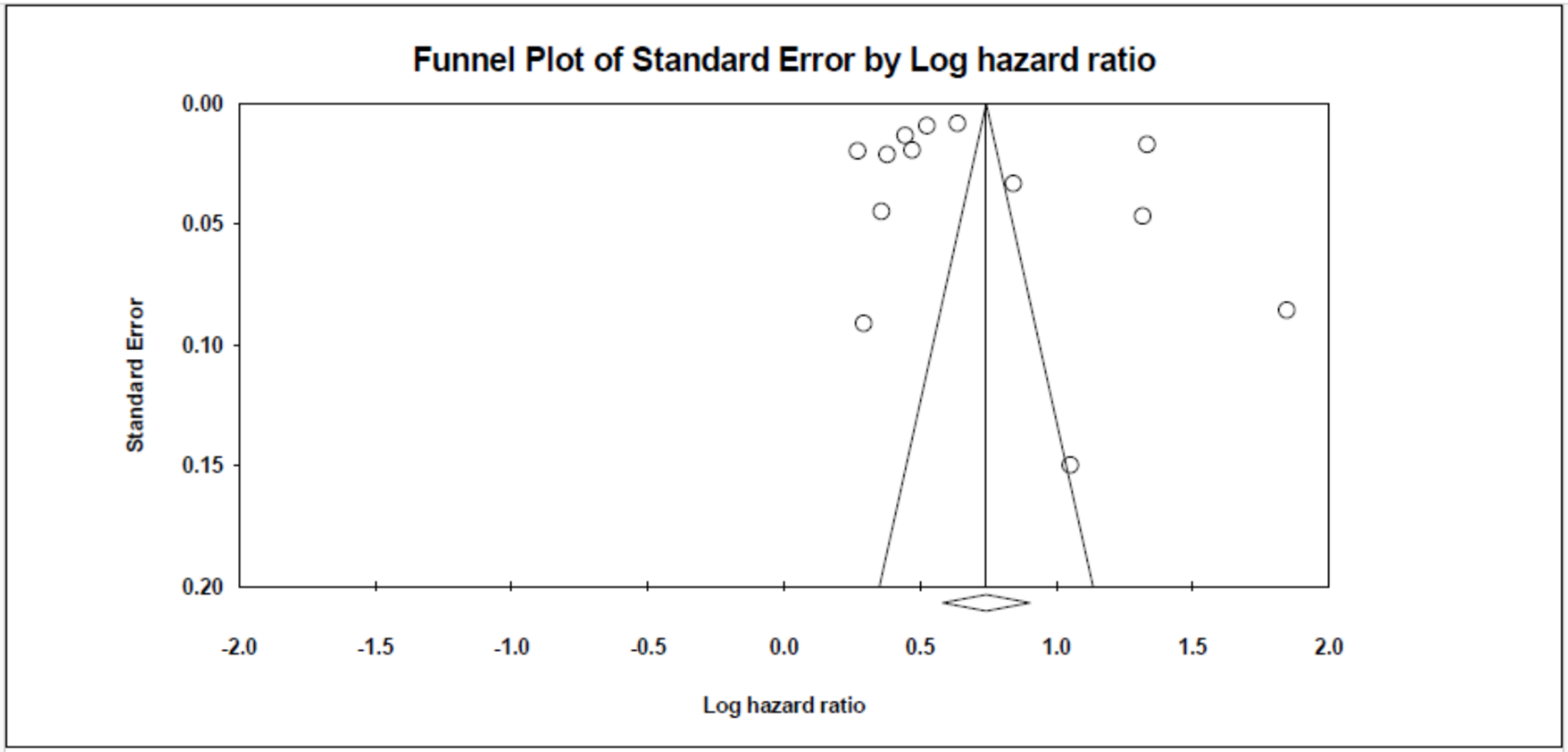

Figure S3

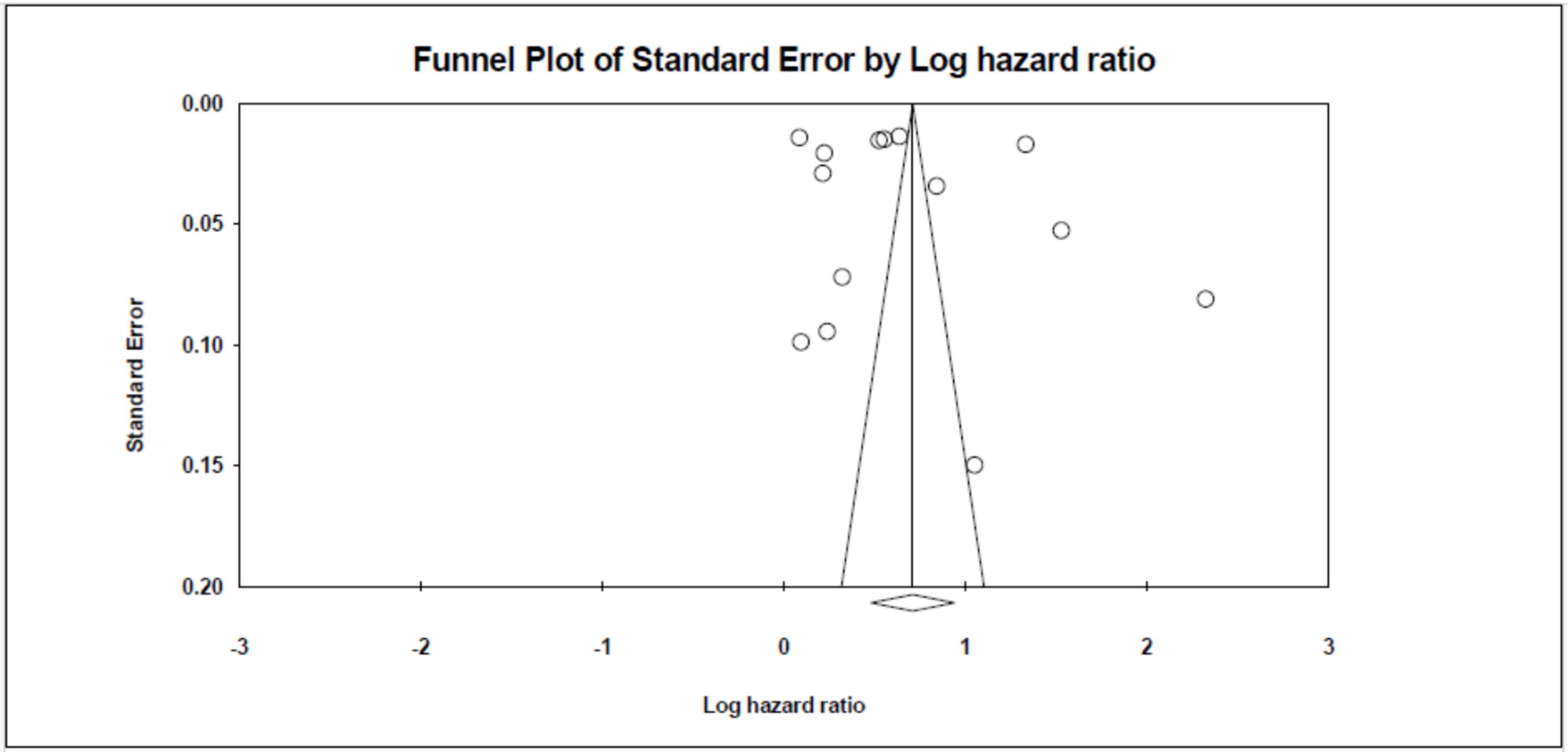

Table S1. Summary of the used search strategy and results.

Source and search date	Search strategy	Results
**PubMed**<br>(NLM)<br><br>**Search date:**<br>2024-10-10	("Traumatic brain injury" OR "TBI" OR "Head trauma" OR "Head injuries" OR "Concussion") AND ("Stroke" OR "Cerebrovascular accident" OR "Brain vascular accident" OR "Brain ischemia" OR "Non-ischemic stroke" OR "Ischemic stroke") AND ("Risk factors" OR "Indicators" OR "Incidence" OR "Association" OR "Predictors")	**1238**
**Cochrane Library**<br><br>**Search date:**<br>2024-10-10	(("Traumatic brain injury" OR "TBI" OR "Head trauma" OR "Head injuries" OR "Concussion") AND ("Stroke" OR "Cerebrovascular accident" OR "Brain vascular accident" OR "Brain ischemia" OR "Non-ischemic stroke" OR "Ischemic stroke") AND ("Risk factors" OR "Indicators" OR "Incidence" OR "Association" OR "Predictors")):ti,ab,kw"	**388**
**Web of Science**<br>(Core collection, Clarivate)<br><br>**Search date:**<br>2024-10-10	ALL=(("Traumatic brain injury" OR "TBI" OR "Head trauma" OR "Head injuries" OR "Concussion") AND ("Stroke" OR "Cerebrovascular accident" OR "Brain vascular accident" OR "Brain ischemia" OR "Non-ischemic stroke" OR "Ischemic stroke") AND ("Risk factors" OR "Indicators" OR "Incidence" OR "Association" OR "Predictors"))	**2891**

**Scopus**<br>(Elsevier)<br><br>**Search date:**<br>2024-10-10	TITLE-ABS-KEY (("Traumatic brain injury" OR "TBI" OR "Head trauma" OR "Head injuries" OR "Concussion") AND ("Stroke" OR "Cerebrovascular accident" OR "Brain vascular accident" OR "Brain ischemia" OR "Non-ischemic stroke" OR "Ischemic stroke") AND ("Risk factors" OR "Indicators" OR "Incidence" OR "Association" OR "Predictors"))	**3782**
Total number of records identified		**8299**
Total number of unique records after removing duplication		**5750**

Table S2. Quality assessment of cohort studies using NOS tool.

Study ID	Type of study	Selection					Comparability	Outcome				Overall	Overall quality
		D1	D2	D3	D4	Overall selection		D5	D6	D7	Overall outcome		
Albrecht 2015	Cohort	1	1	0	1	3	0	1	1	1	3	6	Fair quality
Burke 2013	Cohort	1	1	1	1	4	3	1	0	1	2	9	Good quality
Chen 2011	Cohort	1	1	1	1	4	3	1	1	1	3	10	Good quality
Choi 2024	Cohort	1	1	1	1	4	3	1	1	1	3	10	Good quality
Elser 2024	Cohort	1	1	1	1	4	3	1	1	1	3	10	Good quality
Eric Nyam 2019	Cohort	1	1	1	1	4	3	1	1	1	3	10	Good quality
Izzy 2022	Cohort	1	1	1	1	4	3	1	1	1	3	10	Good quality
Lee 2014	Cohort	1	1	1	1	4	3	1	1	1	3	10	Good quality
Liao 2014	Cohort	1	1	1	1	4	3	1	1	1	3	10	Good quality
Liu 2017	Cohort	1	1	1	0	3	3	1	1	1	3	9	Good quality
McFarlane 2019	Cohort	1	0	1	1	3	0	0	1	1	2	5	Fair quality
Schneider 2023	Cohort	1	1	1	1	4	3	1	1	1	3	10	Good quality
Stewart 2022	Cohort	1	1	1	1	4	3	1	1	1	3	10	Good quality

D1: Is the case definition adequate/Representative of the exposed cohort?
D2: Representative of the cases/Selection of the non-exposed cohort.
D3: Selection of Controls/Ascertainment of exposure.
D4: Definition of Controls/ Demonstration that outcome of interest was not present at start of study.
D5: Ascertainment of exposure/ Assessment of outcome.
D6: Same method of ascertainment for cases and controls/ Was follow-up long enough for outcomes to occur.
D7: Non-Response rate/ Adequacy of follow up of cohorts.

Table S3. GRADE assessment

Certainty assessment							Summary of findings		Importance
**Number of studies**	**Study design**	**Risk of Bias**	**Inconsistency**	**Indirectness**	**Imprecision**	**Others**	**HR (95% CI)**	**Certainty**	
**The pooled stroke risk after TBI based on stroke type**									
4 (All strokes)	Observational	Not serious	Not serious	Not serious	Not serious	None	2.3 (1.7-2.95)	Low	Important
2 (Hemorrhagic stroke)	Observational	Not serious	Not serious	Not serious	Not serious	None	4.8 (3.34-6.94)	Low	Important
7 (Ischemic stroke)	Observational	Not serious	Not serious	Not serious	Not serious	None	1.56 (1.29-1.9)	Low	Important
**The pooled stroke risk after TBI based on stroke Severity**									
7 (All TBI severity)	Observational	Not serious	Not serious	Not serious	Not serious	None	2.24 (1.63-3.1)	Low	Important
4 (Mild TBI)	Observational	Not serious	Not serious	Not serious	Not serious	None	1.8 (1.17-2.8)	Low	Important
3 (Moderate to severe)	Observational	Serious	Not serious	Not serious	Not serious	None	3.64 (2.16-6.14)	Very low	Important
**The pooled stroke risk after TBI based on post-TBI phase**									
2 (0 to 30 days)	Observational	Serious	Not serious	Not serious	Not serious	None	4.15 (2.25-7.7)	Very low	Important
4 (30 days to 1 year)	Observational	Not serious	Not serious	Not serious	Not serious	None	1.7 (1.1-2.6)	Low	Important
8 Greater than 1 year	Observational	Not serious	Not serious	Not serious	Not serious	None	1.9 (1.4-2.54)	Low	Important
